# Xylazine in the Opioid Epidemic: A Systematic Review of Case Reports and Clinical Implications

**DOI:** 10.7759/cureus.36864

**Published:** 2023-03-29

**Authors:** Shahana Ayub, Shanli Parnia, Karuna Poddar, Anil K Bachu, Amanda Sullivan, Ali M Khan, Saeed Ahmed, Lakshit Jain

**Affiliations:** 1 Psychiatry, Cornerstone Family Healthcare, Newburgh, USA; 2 Internal Medicine, Cimpar, Oak Park, USA; 3 Psychiatry, Thomas Jefferson University Hospital, Philadelphia, USA; 4 Psychiatry, Baptist Health University of Arkansas for Medical Sciences, North Little Rock, USA; 5 Psychiatry, Quinnipiac University, Hamden, USA; 6 Psychiatry, University of Texas Rio Grande Valley, Harlingen, USA; 7 Psychiatry and Behavioral Sciences, Rutland Regional Medical Center, Rutland, USA; 8 Psychiatry and Behavioral Sciences, Nassau University Medical Center, East Meadow, USA; 9 Behavioral Health Sciences, Boston University School of Medicine, Boston, USA; 10 Psychiatry, University of Connecticut, Hartford, USA

**Keywords:** case report synthesis, a systematic review, overdose death, fatal outcome, naloxone, co-administration, intravenous injection, adulterant, opioid overdose, xylazine

## Abstract

Introduction and objectives: The opioid overdose epidemic is exacerbated by the emergence of Xylazine as an illicit drug adulterant. Xylazine, a veterinary sedative, can potentiate opioid effects while also causing toxic and potentially fatal side effects. This systematic review aims to assess the impact of Xylazine use and overdoses within the opioid epidemic context. Method: A systematic search was conducted following PRISMA guidelines to identify relevant case reports, and case series related to Xylazine use. A comprehensive literature search included databases like Web of Science, PubMed, Embase, and Google Scholar, utilizing keywords and Medical Subject Headings (MeSH) terms related to Xylazine. Thirty-four articles met the inclusion criteria for this review.

Results: Intravenous (IV) administration was a common route for Xylazine use among various methods, including subcutaneous (SC), intramuscular (IM), and inhalation, with overall doses ranging from 40 mg to 4300 mg. The average dose in fatal cases was 1,200 mg, compared to 525 mg in non-fatal cases. Concurrent administration of other drugs, primarily opioids, occurred in 28 cases (47.5%). Intoxication was identified as a notable concern in 32 out of 34 studies, and treatments varied, with the majority experiencing positive outcomes. Withdrawal symptoms were documented in one case study, but the low number of cases with withdrawal symptoms may be attributed to factors such as a limited number of cases or individual variation. Naloxone was administered in eight cases (13.6%), and all patients recovered, although it should not be misconstrued as an antidote for Xylazine intoxication. Of the 59 cases, 21 (35.6%) resulted in fatal outcomes, with 17 involving Xylazine use in conjunction with other drugs. The IV route was a common factor in six out of the 21 fatal cases (28.6%).

Conclusion: This review highlights the clinical challenges associated with Xylazine use and its co-administration with other substances, particularly opioids. Intoxication was identified as a major concern, and treatments varied across the studies, including supportive care, naloxone, and other medications. Further research is needed to explore the epidemiology and clinical implications of Xylazine use. Understanding the motivations and circumstances leading to Xylazine use, as well as its effects on users, is essential for developing effective psychosocial support and treatment interventions to address this public health crisis.

## Introduction and background

Xylazine, a veterinary tranquilizer, first identified in Puerto Rico as a more prevalent additive in street drugs in the early 2000s, is spreading to other states in the United States at an alarming rate [1]. It is commonly added to opioids to boost their depressive effects on the central nervous system. Besides opioids, it is also common for individuals who abuse cocaine to be exposed to Xylazine, as it is frequently added to illicit substances as an adulterant [2,3].

Xylazine is not approved for human use and is primarily used in veterinary settings as a sedative, analgesic, and muscle relaxant. However, Xylazine is available over the internet to purchase. The easy availability and low cost of Xylazine online have made it an attractive "cutting agent" for drug traffickers [2]. Xylazine's sedative effects allow them to decrease the amount of fentanyl or heroin in drug mixtures while still producing similar effects, making it a profitable addition [2]. In fact, some people intentionally seek out Xylazine-laced fentanyl or heroin as Xylazine is believed to increase the duration of action of fentanyl and heroin.

Its use has been linked to an increase in overdose deaths and side effects including skin ulcers, abscesses, lesions, drowsiness, amnesia, hypotension, bradycardia, and bradypnea [4], making it a significant public health concern [2,5]. Since Xylazine is not an opioid and unfortunately its sedative effects are not reversed by naloxone, an opioid overdose involving Xylazine is much more challenging to reverse and, consequently, more lethal to the user [2,5].

The State Unintentional Drug Overdose Reporting System (SUDORS) provides comprehensive data on opioid overdose deaths and quickly identifies newer, dangerous drugs [6]. According to SUDORS, Xylazine is emerging as an adulterant in illicit drug mixtures, exacerbating the opioid overdose crisis and resulting in opioid overdose deaths in numerous states and cities across the United States, including Vermont, Maine, Massachusetts, Connecticut, Maryland, Pennsylvania (specifically Philadelphia), and New York (specifically New York City) [1,7-13]. In a recent study by Friedman and colleagues (2022), they discovered that Xylazine was present in ten jurisdictions across all four U.S. census regions. Among these locations, Philadelphia had the highest prevalence of Xylazine-related deaths (25.8%), followed by Maryland (19.3%) and Connecticut (10.2%) [1].

One of the coauthors (S-AH) of this research paper, who works at an Opioid Treatment Program (OTP) clinic in Vermont, has observed a rise in the number of patients testing positive for Xylazine in their urine. Official figures from Vermont also reveal a dramatic increase in Xylazine presence in overdose cases since 2020. In the first quarter of 2022, Xylazine was found in 30% of fatal opioid overdoses, and the percentages were even higher in specific months: 50% in February 2020, 41% in June 2022, and 32% in November 2022 [7]. Similarly, within New England, three other states have been heavily impacted as well. In Connecticut, for example, Xylazine-related drug overdose deaths are on the rise. There were 71 deaths in 2019, 141 in 2020, 295 in 2021, and 347 in 2022 [8]. In Massachusetts, Xylazine started showing up in opioid-related overdose deaths around June 2022. By the second quarter of that year, it accounted for 5% of such deaths in Maine, fatal opioid drug overdoses increased to 643 deaths in the first 10 months of 2022, with Xylazine involved in 6 to 8% of the deaths [10].

In Philadelphia, Xylazine went from being detected in less than 2% of cases of fatal heroin and/or fentanyl overdose between 2010 and 2015, to 262 (31%) of the 858 fatal heroin and/or fentanyl overdose cases in 2019 [13]. According to Philadelphia Department of Public Health reports, in 2021, 91% of samples of purported heroin or fentanyl from Philadelphia also contained Xylazine, making it the most common adulterant in the drug supply [11]. Due to the growing number of Xylazine-containing substance confiscations in 2021, all opioid samples began to be tested for Xylazine. Because of this, Xylazine was found in 429 overdose deaths (19% of opioid-related overdose deaths), compared to 52 overdose deaths (3% of opioid-related overdose deaths) in 2020 [12]. The National Forensic Laboratory Information System's data supports these trends, showing a significant rise in Xylazine detection in drug items and cases analyzed by forensic labs over time [5].

Several agencies at National and State levels, including the Drug Enforcement Administration (DEA), Food and Drug Administration (FDA), Centers for Disease Control and Prevention (CDC), and state public health departments, have put out warnings about Xylazine since it was found to be more common and to have a serious impact on opioid overdose deaths. Xylazine is still legal in the U.S., but the FDA has taken steps to prevent the drug from entering the U.S. market for illicit purposes on February 28, 2023 [14]. The aim of these measures is to limit the illicit use of Xylazine while ensuring it remains available for legitimate veterinary purposes. Moreover, various state health agencies have separately warned about the risks associated with Xylazine-contaminated drugs in their regions [15]. In this review, we aimed to explore the impact of Xylazine use in exacerbating the opioid overdose epidemic. We also discussed the pharmacologic properties that contribute to Xylazine's potential lethality and reviewed the available data on treatment options.

## Review

Methods

This review was conducted following the Preferred Reporting Items for Systematic Reviews and Meta-Analyses (PRISMA) guidelines. We aimed to identify all relevant studies, including case reports and case series related to Xylazine use, its clinical features, and management. Due to the limited number of randomized controlled trials (RCTs) or clinical trials on this topic, our primary emphasis was on case reports and case series.

Search strategy

We conducted a comprehensive literature search using electronic databases, including Web of Science, PubMed, Embase, Google Scholar, and gray literature sources, such as conference proceedings and government reports, from inception until the present date. Our search strategy used a combination of keywords and Medical Subject Headings (MeSH) terms related to "xylazine*," "xylazine use," xylazine overdose, "xylazine intoxication," "xylazine withdrawal,” and "xylazine management." This search yielded 1,238 articles, of which 34 met the inclusion criteria. Two independent reviewers (SA and LJ) screened the titles and abstracts of identified articles for eligibility. Full-text articles were obtained for potentially relevant studies, and eligibility was determined based on the following inclusion criteria: (1) studies reporting on Xylazine use; (2) human case reports and case series; (3) articles published in English. Studies were excluded if they (1) did not focus on Xylazine use or were not relevant to the objectives of this review. Disagreements between reviewers were resolved by consensus or by consulting a third reviewer (AB).

Study selection

**Figure 1:**
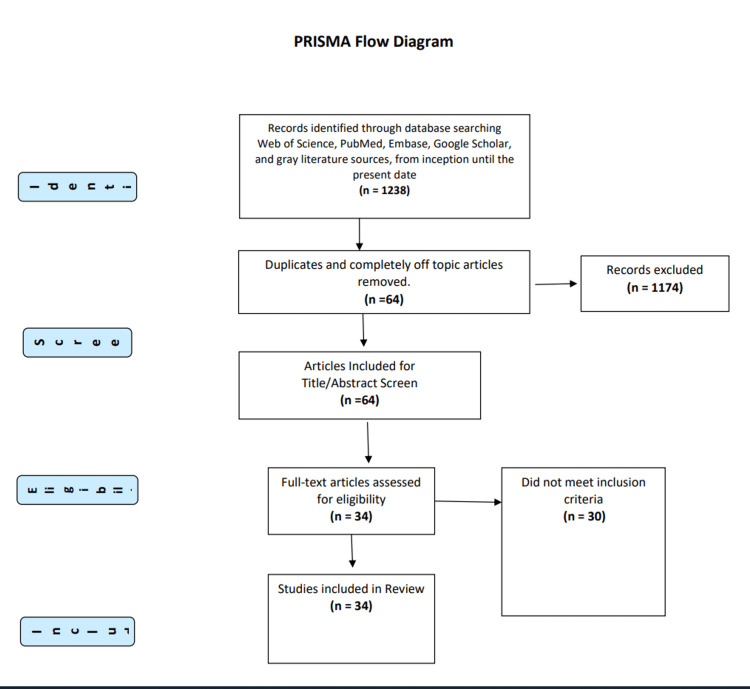
PRISMA Diagram PRISMA: Preferred Reporting Items for Systematic Reviews and Meta-Analyses

A total of 1,238 articles were identified through the initial database search. After removing duplicates and screening titles and abstracts, 64 articles were selected for full-text review. From these, 34 articles met the inclusion criteria and were included in this systematic review. These articles encompassed 59 cases involving Xylazine use for recreational purposes, self-harm, or accidental exposure (Figure 1).

Quality assessment

Since there were not any controlled studies, we gauged the evidence quality by examining the reliability and validity of the public sources used in this review. We considered the relevance, reliability, and precision of these sources, discussing any potential biases we came across. Additionally, we evaluated the evidence quality by comparing the consistency of findings across different sources.

Studies included

Case studies included: Ehrman-Dupre et al. [16], Stillwell et al. [17], Elejalde et al. [18], Liu CM et al. [19], Deutsch SA et al. [20], Andreson-Streicht et al. [21], Mulders et al. [22], Bayramoglu A et al. [23], Spoerke et al. [24], Carruthers et al. [25], Samanta et al. [26], Ganapathy et al. [27], Shapses et al. [28], Miller et al. [29], Hoffman et al. [30], Velez et al. [31], Krongvorakul et al. [32], Gallanosa et al. [33], Capraro et al. [34], Wolowich et al. [35], Meyer et al. [36], Poklis et al. [37], Arican et al. [38], Mccloskey et al. [39], Snow et al. [40], Choon et al. [41], Ramon et al. [42], Lewis et al. [43], Haigh et al. [44], Wong et al. [45], Chavez et al. [46], Barroso et al. [47], Briellman et al. [48], and Mittelman et al. [49]. See Table 1.

**Table 1:** Cases Included in Review IV: intravenous; IM: intramuscular; SC: subcutaneous; PCA: patient-controlled analgesia; PICU: pediatric intensive care unit

Study	Author(s)	Number of cases each study	Accidental/Intentional/Abuse	Xylazine Use Route	Xylazine dose	Xylazine Use and other drug involved (concomitant use of drugs)	Intoxication	Withdrawal	Treatment/intervention/management	Outcome/sequelae	
1	Ehrman-Dupre et al. [16]	1	Abuse	IV	na	Tramadol, Fentanyl	no	yes	Day 1 - Dexmedetomidine + Tizanidine; Day 2 Phenorbarbital + Clonidine; Hydromorphone PCA + Gabapentin + Ketamine for pain; Buprenorphine Microinduction	Recovered	
2	Stillwell et al. [17]	1	na	IM	450mg	Paroxetine	yes	na	na	na	
3	Elejalde et al. [18]	1	na	Inhalation	na	na	yes	no	IV fluids only	Recovered	
4	Liu CM et al. [19]	1	Abuse	Inhalation	na	Sulpiride, Ketamine, Phenobarbital	yes	no	IV fluids only	Recovered	
5	Deutsch et al. [20]	3	Accidental	na	na	Case 1 - Morphine and Fentanyl; Case 2 and 3 Fentanyl	yes	no	Case 1 - Naloxone (nasal+IV), Intubation, PICU; Case 2 - IV Naloxone; Case 3 - Intubation	Recovered	
6	Andreson-Streicht et al. [21]	1	Intentional	IM	na	na	yes	no	Supportive Treatment	Recovered	
7	Mulders et al. [22]	1	Abuse	SC	500mg	na	no	no	Clonidine	Neurocognitive Symptoms	
8	Bayramoglu et al. [23]	1	Intentional	IV	500mg	na	yes	no	Defibrillation	Fatal	
9	Spoerke et al. [24]	3	Intentional (1), na (2)	IV (1); IM (2)	40mg, 2400mg	na	yes	no	Naloxone	Recovered	
10	Carruthers et al. [25]	1	Intentional	IV	900mg	na	yes	no	Lidocaine; Supportive Treatment	Recovered	
11	Samanta et al. [26]	1	Accidental	SC	200mg	na	yes	no	Naloxone	Recovered	
12	Ganapathy et al. [27]	1	Accidental	IM	na	na	yes	no	Supportive Treatment	Recovered	
13	Shapses et al. [28]	1	Abuse	na	na	Fentanyl, Cocaine	yes	no	Supportive Treatment, Cardiac Catheterization	Recovered	
14	Miller et al. [29]	1	Accidental	na	na	Fentanyl	yes	no	Supportive Treatment	Recovered	
15	Hoffman et al. [30]	1	Intentional	IM	1500mg	na	yes	no	Supportive Treatment + Etomidate, Propofol	Recovered	
16	Velez et al. [31]	1	Accidental	Ocular	800mg	na	yes	no	Supportive Treatment	Recovered	
17	Krongvorakul et al. [32]	3	Intentional	Ingestion	na	na	yes	no	Supportive Treatment	Recovered	
18	Gallanosa et al. [33]	1	Intentional	Ingestion	400mg	na	yes	no	IV Naloxone + Intubation	Recovered	
19	Capraro et al. [34]	1	Abuse	Inhalation	4300mg	Benzodiazepines	yes	no	IV Naloxone + Intubation	Recovered	
20	Wolowich et al. [35]	1	Intentional	IM	2500mg	na	yes	no	Supportive Treatment	Recovered	
21	Meyer et al. [36]	1	Accidental	Arrow Injection (IM)	na	Ketamine	yes	no	Supportive Treatment	Recovered	
22	Poklis et al. [37]	1	Intentional	IV	na	Chlorazepate, Alcohol	yes	no	na	Fatal	
23	Arican et al. [38]	1	Abuse	IV	1500mg	Ketamine (1000mg)	yes	no	IV fluids, Metoprolol	Recovered	
24	Mccloskey et al. [39]	1	Intentional	Ingestion	120mg	Cannabinoids	yes	no	IV Naloxone + IV Atropine + Supportive Treatment	Recovered	
25	Snow et al. [40]	1	Intentional	IV	1000mg	na	yes	no	Supportive Treatment	Recovered	
26	Choon et al. [41]	2	Accidental	IM	na	na	yes	no	Supportive Treatment + IV Atropine in both; Case 1 - Oxygen, Case 2 IV Naloxone and Noradrenaline	Recovered	
27	Ramon et al. [42]	1	Abuse	Inhalation	na	na	yes	na	na	na	
28	Lewis et al. [43]	1	Abuse	IM	na	na	yes	no	na	na	
29	Haigh et al. [44]	4	Abuse (1); Accidental (2-4)	IV (1), Ingestion (2) SC (1)	na	na	yes	no	Case 1 -Supportive Treatment; Case 2 and 3 - 24 hrs Observation; Case 4 No Treatment	Recovered	
30	Wong et al. [45]	7	Abuse	IV (suspected in 2 cases 4,6) remaining 5 na	na	7 Cases Fentanyl; 6 Cases Heroin; 5 Cases Morphine; 5 Cases Codeine; 5 Cases Cocaine; 4 Cases Alcohol; 4 Cases Procaine; 3 Cases Lidocaine; 3 Cases Quinine/Quinidine; 2 Cases Alprazolam; 2 Cases Diltiazem; 2 Cases Naproxen; 1 Case - PCP, Diphenhydramine, Ibuprofen, Citalopram, Mirtazapine, Hydroxyzine	yes	no	na	Fatal	
31	Chavez et al. [46]	9	Abuse	IV (suspected in 2 cases); remaining 7 na	na	8 Cases Morphine; 6 cases Cocaine; 5 Cases Heroin; 4 Cases Alcohol; 1 Case Codeine	yes	no	na	Fatal	
32	Barroso et al. [47]	1	Accidental	SC	na	na	yes	no	Supportive Treatment	Recovered	
33	Briellman et al. [48]	1	Intentional	na	na	na	yes	na	na	Fatal	
34	Mittelman et al. [49]	2	Intentional	na	na	na	yes	na	na	Fatal	

Literature review

The literature review consists of Xylazine exposure, risks, consequences, mechanism of action, and clinical management in humans (evidence from human and animal studies).

FDA warning

The FDA has warned healthcare professionals about the serious risks associated with Xylazine exposure in humans. Xylazine is primarily used as a veterinary anesthetic and has no approved uses for humans [15]. Xylazine is increasingly found in the illicit drug supply, often in combination with other drugs. Acute and repeated exposure to Xylazine can lead to significant harm, including delayed diagnosis and management of polysubstance overdose, interference with the successful treatment of opioid use disorder (OUD), and the development of severe, necrotic skin ulcerations [15].

Mechanism of action

Xylazine acts as an 𝞪2-receptor agonist in both central and peripheral nervous systems, causing a strong sympatholytic effect by activating central presynaptic 𝞪2 receptors [50]. It was originally synthesized with the goal of creating a new anti-hypertensive drug due to its similarity to clonidine. Its strong CNS depressant effects led to it being used as a veterinary sedative, analgesic, and muscle relaxant in the 1960s [51].

Pharmacokinetics and overdose

Xylazine has a rapid elimination from the body, with a half-life ranging from 23 to 50 minutes [15], posing a significant challenge in the management of Xylazine overdose cases. Xylazine is a veterinary drug, and there is limited information available on its pharmacokinetics and clinical management in cases of human overdose. Its rapid metabolism and elimination from the body can lead to a quick onset of toxic effects, potentially overwhelming the patient's system before appropriate interventions can be implemented. Timely identification and treatment of this substance are critical in managing Xylazine overdose cases.

Toxicity and fatality

The published literature demonstrates that Xylazine can produce toxicity and fatality in humans in doses ranging from 40 to 2400 mg, with plasma concentrations ranging from 0.03 to 4.6 mg/L in non-fatal cases. In fatalities, blood concentrations of Xylazine range from trace to 16 mg/L [52]. Due to the significant overlap between non-fatal concentration and postmortem blood concentration, there appears to be no defined safe, toxic, or fatal concentration of Xylazine in humans [50]. It is important to note that comprehensive pharmacokinetic data examining various routes of administration (IV, IM, inhalation) and their impact on bioavailability and fatal dosages are limited. This lack of comprehensive pharmacokinetic data may have an impact on the interpretation of the reported results, and readers should take this into account when assessing the findings presented in this study.

Health consequences and clinical management

In humans, Xylazine usage has been linked to damage to multiple organs resulting in bradycardia, elevated blood sugar, hypotension, and even coma in an overdose. Although naloxone can be used to reverse opioid overdose, the literature indicates that it is not an effective medication for Xylazine overdose [30]. As a result, other supportive treatments must be provided to patients not responsive to naloxone therapy. Additionally, repeated exposure to Xylazine has been found to cause characteristic necrotic skin ulcers in patients. The exact mechanism of skin injury is not fully understood.

Complicating polysubstance intoxication management

As Xylazine is frequently found mixed with other substances, it can significantly impact the clinical management of acute intoxication/withdrawal of other substances. While its interaction with opioids is described above; Xylazine was also shown to impair the anticonvulsant properties of phenobarbital, phenytoin, and diazepam in rats [53]. Thus, Xylazine can impede the clinical treatment of withdrawal seizures and increase morbidity and mortality.

Abuse potential

Xylazine has been identified as a substance with significant abuse potential. Xylazine combined with other drugs intensifies its sedative effects and can be dangerous. The exact mechanism of Xylazine abuse is unclear, but it may involve its impact on the brain's reward system through altering neurotransmitter levels, such as norepinephrine and dopamine [5]. This alteration in neurotransmitter levels may produce pleasurable effects that lead to addiction. Studies published from Puerto Rico and Philadelphia indicate that it is commonly found as a mixed substance with speedball, a combination of stimulants and opioids. In other cases, it has been detected with heroin, fentanyl, and cocaine [50]. According to a report published in Puerto Rico, Xylazine was found in more than 90% of the speedball samples [54]. In light of these concerning findings, more attention and awareness are required in combating Xylazine abuse and developing specific interventions against it.

Hemodynamic effects

Xylazine exposure has been associated with various cardiovascular and pulmonary effects, as well as challenges in managing hypotension and diuresis. Several studies and case reports emphasize the cardiac complications that can arise from Xylazine use, such as biventricular systolic failure, valvular dysfunction, and myocardial necrosis and fibrosis [55-57]. In some cases, clinical treatment with nifedipine has been found effective in managing these cardiac complications [58]. In addition to its impact on the cardiovascular system, Xylazine has been linked to pulmonary issues. For example, a study conducted by Chavez et al. on nine Xylazine-related deaths in Puerto Rico discovered moderate to severe pulmonary congestion and edema in all cases. This could be attributed to either a direct effect of Xylazine on the pulmonary vasculature or as a consequence of the drug's impact on cardiac activity [46]. When it comes to managing Xylazine-induced hypotension and diuresis, many patients respond well to intravenous (IV) fluids alone, without the need for additional interventions [19,20]. Animal studies have demonstrated that Xylazine can cause diuresis, which can be reversed by Atipamezole and yohimbine [59].

Effects on pregnancy

While no human studies are currently available to establish the safety of Xylazine use during pregnancy, findings from animal studies raise concerns about its potential adverse effects on fetal development [60,61]. In these studies, Xylazine has been shown to markedly reduce uterine blood flow and oxygen availability, which may critically impair the delivery of oxygen to the fetus during crucial stages of development or delivery.

Hyperglycemia and other long-term effects

Xylazine use in animals can lead to hyperglycemia, induced in normoglycemic and insulin-dependent diabetic monkeys via reducing tissue sensitivity to insulin and glucose uptake [62]. A metabolite of Xylazine, 2,6-xylidine, is also reported to be a genotoxic and carcinogenic compound. These can create challenges for people who use Xylazine chronically [63].

Ocular damage

The use of Xylazine as anesthesia led to a decrease in intraocular pressure after intramuscular (IM) administration in monkeys [64] and corneal lesions after IV administration in rats [65].

Table 2 provides the frequency of variables.

**Table 2:** Frequency of Variables IV: intravenous; IM: intramuscular; SC: subcutaneous

Variable	Count	Description
IV route	12 (8 confirmed, 4 suspected)	Cases of Xylazine use via intravenous injection
IM route	9	Cases of Xylazine use via intramuscular injection
Inhaled route	4	Cases of Xylazine use via inhalation
Ingested route	5	Cases of Xylazine use via ingestion
SC route	4	Cases of Xylazine use via subcutaneous injection
Ocular route	1	Cases of Xylazine use via ocular route
Arrow injection (IM)	1	Cases of Xylazine use via arrow injection (intramuscular)
Not specified route	15	Cases with unspecified route of Xylazine use
Intoxication	57	Cases with Xylazine intoxication
Withdrawal	1	Cases with Xylazine withdrawal
No Withdrawal, No Intoxication	1	Cases with no withdrawal, and no intoxication
Recovered outcome	34	Cases with a recovered outcome
Fatal outcome	21	Cases with a fatal outcome
Not specified outcome	3	Cases with unspecified outcome
Accidental use	14	Cases of accidental Xylazine use
Intentional use	16	Cases of intentional Xylazine use
Abuse	25	Cases of Xylazine abuse

Table 3 provides naloxone usage, cases of abuse, and co-administration of other drugs.

**Table 3:** Naloxone Usage, Cases of Abuse, and Co-administration of Other Drugs

Variable	Count	Description
Naloxone used	8	Cases where naloxone was used as treatment for Xylazine intoxication
Abuse	25	Cases of Xylazine abuse
Opioids (E.g. Fentanyl, Morphine, Heroin co-administration)	21	Cases where Xylazine was used alongside Fentanyl, Morphine, Heroin, Tramadol
Ketamine co-administration	3	Cases where Xylazine was used alongside ketamine
Cocaine co-administration (speedball cases included)	12	Cases where Xylazine was used alongside cocaine
Cannabis and Benzodiazepines	3	Cases where Cannabis and Benzodiazepines

Results

Route of Administration, Dose, and Outcomes

Doses ranged from 40 mg to 4300 mg, with many cases not reporting specific dosages. The average dose of Xylazine in fatal cases was 1,200 mg, compared to 525 mg in non-fatal cases, suggesting that higher doses might be associated with an increased risk of fatal outcomes. However, it is important to consider individual cases' details when interpreting these findings. Out of the 12 IV Xylazine use cases, two were fatal, and the rest recovered. However, it's important to consider the concomitant use of other drugs, which might have affected the outcomes. In the fatal IV cases, no other drugs were involved in the Bayramoglu et al. case, while in the Poklis et al. case, clorazepate and alcohol were also involved. While the average dose in fatal cases is higher than in non-fatal cases, individual case outcomes do not always follow this pattern. Two case studies reported by Carruthers et al. and Hoffman et al. have shown that patients recovered after receiving high doses of Xylazine through IV and IM routes, respectively. However, while there is some evidence suggesting a link between higher doses of Xylazine and fatal outcomes, the relationship is complex and difficult to establish definitively based on the limited data available.

Co-administration of Other Drugs

In a review of 34 studies encompassing a total of 59 cases, it was found that 28 instances (47.5%) involved the concurrent administration of other drugs in combination with Xylazine. Of these cases, the majority involved the use of opioids, including fentanyl, morphine, and heroin, which highlights the potential exacerbation of the ongoing opioid crisis due to the misuse of Xylazine. Notably, several studies, including Ehrman-Dupree et al., Deutsch et al., Shapses et al., and Miller et al., reported the concomitant use of fentanyl, while Wong et al. reported the use of morphine, heroin, fentanyl, and codeine in seven cases. Chavez et al. also reported the use of morphine, alcohol, and cocaine in several cases.

Non-opioid substances, including benzodiazepines, ketamine, and cocaine, were reported in various cases, potentially adding to the complexity of clinical presentation and management of Xylazine overdose. Among the analyzed cases, Capraro et al. reported the use of benzodiazepines; Liu CM et al. cited sulpiride, ketamine, and phenobarbital; and Shapses et al. identified cocaine. The co-administration of Xylazine with other drugs, particularly opioids, intensifies the challenges of managing intoxication and may lead to adverse outcomes.

Naloxone Use and Outcomes

In the context of Xylazine intoxication, the use of naloxone, an opioid antagonist, presents a complex and nuanced picture. We analyzed 59 cases involving Xylazine intoxication, in which eight patients (13.6%) were administered naloxone, and all of them recovered. In cases where Xylazine is used in conjunction with opioids, naloxone's effectiveness in reversing the effects of opioids may indirectly contribute to patient recovery by mitigating the impact of opioid intoxication. However, in cases of Xylazine intoxication without opioid involvement, naloxone may not be effective, as demonstrated by a case of a 16-year-old patient who showed no response to naloxone (Capraro et al.). It should be noted that observing the reversal of a Xylazine overdose with naloxone should not be taken as an indication that naloxone is a specific antidote for Xylazine intoxication. Further studies are needed to confirm the effectiveness of naloxone in a broader range of scenarios involving Xylazine intoxication and investigate its potential role in treating such cases, particularly when used in combination with other substances.

Xylazine Abuse and Outcomes

In our analysis of Xylazine abuse, we examined 34 studies, out of which 11 reported a total of 25 cases of Xylazine abuse. The cases varied in terms of administration routes, concomitant drug use, and outcomes. We observed that the most common route of Xylazine administration was IV, followed by inhalation, IM, and subcutaneous (SC). A significant number of cases involved the concomitant use of other drugs, particularly opioids such as fentanyl, heroin, and morphine. Other substances commonly reported in combination with Xylazine included cocaine, codeine, benzodiazepines, and ketamine. Out of the 25 Xylazine abuse cases, 16 (64%) resulted in fatal outcomes, emphasizing the severity of Xylazine overdoses in the context of the opioid crisis. The remaining cases had various outcomes, including recovery and the development of neurocognitive symptoms.

Intoxication and Withdrawal Symptoms

Intoxication was identified as a notable concern, as it was reported in 32 out of 34 studies. The treatments administered for intoxication varied among the studies, encompassing supportive care, IV fluids, and specific interventions such as naloxone administration, intubation, and cardiac catheterization. The majority of patients experienced positive outcomes and successful recovery. However, there were instances where intoxication proved fatal, emphasizing the critical need for timely and appropriate treatment in cases of Xylazine overdose. Withdrawal symptoms were documented in one case study, where the patient's withdrawal symptoms and potential for harmful autonomic instability were managed using a combination of medications, including dexmedetomidine infusion, phenobarbital, tizanidine, and clonidine.

It is important to note that the low incidence of withdrawal symptoms may be attributed to factors such as a limited number of cases, overlapping symptoms resulting from the co-use of various drugs during withdrawal (which further complicates the identification of Xylazine's individual withdrawal symptoms), differing methodologies employed by researchers, or the possibility that certain individuals may not experience withdrawal symptoms associated with Xylazine use.

Xylazine-Related Fatal Outcomes

Out of the 59 cases, 21 (35.6%) had fatal outcomes. Among these fatal cases, 17 (81%) involved the use of Xylazine in combination with other drugs, including opioids (e.g., fentanyl, heroin, morphine, and codeine), stimulants (e.g., cocaine), and sedatives (e.g., alcohol, alprazolam, procaine, and lidocaine). The fatal cases reported in this paper involve a wide range of Xylazine doses, with only one case (Bayramoglu et al.) reporting a larger dose of 500 mg. The IV route of administration was reported in six of the 21 fatal cases (28.6%), and in the other 13 cases not reported. It is important to note that IV use was suspected in four of these cases due to indications of a needle found near the decedent or recent venipuncture sites. These findings suggest that the risks of Xylazine use are significantly increased when combined with other drugs, and IV use may have also increased the risk of adverse outcomes, as seen in some of the fatal cases. It is important to note that due to the limited data, establishing a definitive causal relationship between heavy doses of Xylazine and fatal outcomes is challenging.

Discussion

In addressing the growing concerns surrounding Xylazine use, it is crucial to consider the detection, management, and harm reduction strategies to effectively mitigate the adverse effects associated with this substance. Detecting Xylazine in drug samples can be accomplished using methods such as gas chromatography-mass spectrometry (GC-MS) [54]. However, this technique is expensive and time-consuming, prompting the development of alternative approaches like Xylazine test strips, which provide a more cost-effective and rapid means for presumptive testing [66]. Other proposed solutions involve novel electrochemical sensor platforms like a laser-scribed graphene device [67] or an electrochemical paper-based analytical device [68].

The observed association between IV injection and increased risk of fatal outcomes underscores the need for harm reduction strategies that target individuals who use Xylazine via this route. Although no specific harm reduction strategies are present for Xylazine use, harm reduction measures that are generally utilized for IV drug use can be considered. This may include efforts to promote safer injection practices, providing sterile injections or snorting supplies, or the provision of supervised injection facilities [69]. Some researchers suggest smoking or snorting drugs as an alternative to injecting them, with the aim of decreasing the risk of blood-borne infections and other adverse effects; however, it is important to note that this method does not eliminate the possibility of overdose [70,71]. Wound care kits containing antiseptic solutions, bandages, and antibiotic ointments can prevent infections and promote healing in cases of skin abscesses or injection-related injuries [69].

In our study, we have observed some indications that higher doses of Xylazine may be associated with fatal outcomes. However, given that individuals may be unaware of their Xylazine consumption, ensuring dose awareness and providing education to prevent overdose becomes a challenging task. Therefore, it is crucial to consider this complexity when addressing the issue of Xylazine-related overdoses. Given the growing crisis of polydrug overdoses, it is crucial for treatment providers to assess and address concurrent substance use disorders through evidence-based interventions. Although naloxone is ineffective against Xylazine overdose, it should still be provided due to the increasing incidence of concomitant opioid use.

There are no FDA-approved medications for treating Xylazine intoxication or managing Xylazine withdrawal. As Xylazine is not an opioid, its management presents unique challenges. No specific antidotes for Xylazine are approved for human use. Though there are no established guidelines or protocols for treating Xylazine overdose, case studies have documented a variety of approaches. These include the administration of naloxone in the presence of co-occurring opioids, supportive care such as intubation and mechanical ventilation, and the use of medications like clonidine, tizanidine, dexmedetomidine, and others [16,72,73]. However, because Xylazine is often co-used with opioids, naloxone is typically administered as the first step in management [72]. Monitoring of magnesium and potassium, wound care, and other supportive measures are also necessary [72]. According to Dr. D'Orazio, other medications, such as guanfacine, ketamine, gabapentin, pentobarbital, benzodiazepines, some antipsychotics, and lofexidine, can be considered [73]. A case study by Ehrman-Dupre et al. demonstrated the management of Xylazine withdrawal symptoms with dexmedetomidine, tizanidine, and later clonidine and phenobarbital [16]. Opioid use disorder was addressed with buprenorphine.

To reverse Xylazine-induced bradycardia and hypotension, atropine has been used in several case reports [33]. Other potential reversal agents include yohimbine, a potent alpha2-antagonist [74,24] tolazoline [24], and atipamezole, which has shown promising results in animals and was well-tolerated in humans during phase 1 trials [75,76]. Management of skin ulcers caused by Xylazine requires wound care, including debridement techniques, and the use of silver sulfadiazine cream or antibacterial ointments. In severe cases, skin grafting and/or amputation may be considered [72,77].

In our analysis, we observed that a notable portion of cases involved concomitant Xylazine use with other drugs. The majority of these cases involved opioids, such as fentanyl, morphine, and heroin. This observation suggests that the misuse of Xylazine could potentially exacerbate the ongoing opioid crisis. The concomitant use of Xylazine with other drugs, particularly opioids, makes it difficult to manage and treat drug overdose, which may lead to adverse outcomes.

Given the frequent concomitant use of Xylazine use with opioids, we also analyzed data on naloxone treatment of Xylazine poisoning. In our review of case studies, we found that naloxone was given in eight cases, with most of them recovered. It is plausible that the reversal of Xylazine overdose in these cases resulted from naloxone's effectiveness in reversing the effects of opioids and contributed to the overall recovery of these patients. For example, Deutsch et al. reported the successful use of naloxone in treating three patients with Xylazine poisoning, where morphine and fentanyl were also involved [20]. We emphasize that Xylazine is not an opioid, and naloxone is not an established treatment for Xylazine overdose. The use of naloxone in these reported cases should be considered in the context of managing concomitant opioid intoxication and should not be misconstrued as a specific treatment for Xylazine overdose.

## Conclusions

This research paper emphasizes the complex nature of Xylazine use and the urgent need for a comprehensive approach to its detection, management, and harm reduction. The potential association between IV injection, higher Xylazine doses, and fatal outcomes highlights the importance of harm reduction strategies. Moreover, our findings underscore the need to understand the interactions between Xylazine and co-administered substances, particularly in the context of polydrug use involving fentanyl. While naloxone may play a role in treating cases with concurrent opioid intoxication, it is not a specific antidote for Xylazine overdose. Understanding the motivations and circumstances leading to Xylazine use is vital for developing effective psychosocial support and substance use disorder treatment interventions.

Strengths and limitations

This systematic review has several strengths, including a comprehensive search strategy covering multiple databases and gray literature, which increases the likelihood of identifying relevant studies. Incorporating case reports and case series provides a broader understanding of Xylazine use and its implications within the opioid crisis. The detailed analysis of 59 cases sheds light on patterns and risk factors associated with Xylazine-related overdoses and fatalities. By adhering to PRISMA guidelines, the review maintains a robust methodology, enhancing reliability and reproducibility.

However, limitations include a small number of cases, potential publication bias, and heterogeneity in study design and reporting, which could affect generalizability and result interpretation. Establishing causality between identified factors and Xylazine-related overdoses or fatalities requires further research, such as prospective cohort studies or controlled trials.

Important disclaimer

Interpretation and Application of Findings in Clinical Contexts

It is important to acknowledge that this study's results are based on a systematic review of existing literature and should be interpreted with caution in clinical contexts. The findings presented in this review should not be regarded as definitive but as a synthesis of the current evidence on Xylazine use and its clinical implications. As with any literature review, limitations may exist in the included studies, such as potential publication bias, heterogeneity in study design and reporting, and the inability to establish causality. Therefore, clinicians and researchers should carefully consider the evidence presented in this review and weigh it against their knowledge, expertise, and the specific circumstances of their patients before making any clinical decisions or drawing conclusions. Further research, including prospective cohort studies or controlled trials, is needed to confirm the relationships identified in this review and to advance our understanding of Xylazine use and its potential consequences.
